# Caudal Duplication Syndrome: A Case Report Emphasizing Functional Assessment in Surgical Management

**DOI:** 10.7759/cureus.100684

**Published:** 2026-01-03

**Authors:** Rodolfo Jaime Dávila, Adrián Gutiérrez González, José I Cornelio Ramos, Carlos A Ortíz Ruiz, Fernando F Montes Tapia

**Affiliations:** 1 Urology, Hospital Universitario "Dr. José Eleuterio González", Monterrey, MEX; 2 Pediatric Surgery, Hospital Universitario "Dr. José Eleuterio González", Monterrey, MEX

**Keywords:** bladder duplication, case report, caudal duplication syndrome, cystoplasty, diphallia, pediatric urology

## Abstract

Caudal duplication syndrome (CDS) is an extremely rare congenital anomaly involving variable duplication of genitourinary, gastrointestinal, and neural structures. We report a male infant with complete duplication of the bladder, urethra, prostate, penis, scrotum, and colon, associated with a tethered spinal cord and a colovesical fistula. The fistula was not identified on initial imaging and was detected only during intraoperative cystoscopy and cystogram, underscoring the diagnostic limitations of early radiologic evaluation in CDS.

Management was staged and prioritized according to functional urgency. Initial treatment focused on spinal cord detethering and resection of the accessory colon to address neurological risk and prevent recurrent infection. Urologic intervention was subsequently guided by videourodynamic findings, favoring a minimally invasive, function-preserving strategy rather than extensive anatomical resection. After functional stability was achieved, aesthetic genital reconstruction was performed to optimize external genital symmetry and reduce potential psychosocial impact.

This case highlights the highly unpredictable anatomical variability of CDS and reinforces the need for individualized, multidisciplinary management tailored to functional priorities rather than anatomical duplication alone. Long-term follow-up is essential to monitor urinary continence, bladder compliance, and future reproductive potential throughout growth

## Introduction

Caudal duplication syndrome (CDS) is an exceptionally rare congenital anomaly characterized by variable duplication of caudal structures, including elements of the gastrointestinal, genitourinary, and neural systems. Its incidence has been estimated at approximately one in 100,000 live births with a female predominance, although published cases remain scarce and phenotypic expression is highly heterogeneous [1-3]. The embryologic basis involves abnormal division of the cloaca, hindgut, and notochord during early development, resulting in a wide spectrum of anatomical variants without a clearly established etiologic or environmental association [2-4].

Because CDS may simultaneously affect multiple organ systems, management typically requires a tailored, staged, multidisciplinary approach [3,5,6]. Recent reports highlight that treatment algorithms cannot be standardized and should prioritize functional preservation, particularly regarding urinary, gastrointestinal, and neurological outcomes [3,7-9]. Advances in diagnostic modalities, including high-resolution endoscopy, urodynamics, and detailed cross-sectional imaging, have improved early characterization of duplicated structures. Yet, significant anatomical details may still remain undetected in initial evaluation [7-9].

We present an extremely uncommon variant of CDS involving complete duplication of the bladder, urethra, prostate, penis, scrotum, and colon, associated with a tethered cord and a colovesical fistula that was not identified on early imaging. This case provides clinically relevant insight into the diagnostic limitations of conventional studies and illustrates how staging surgical interventions based on functional priorities, rather than anatomical duplication alone, can optimize long-term outcomes. Through correlation with previously reported experiences, we aim to highlight a practical framework for decision-making in complex CDS presentations.

## Case presentation

**Figure 1:**
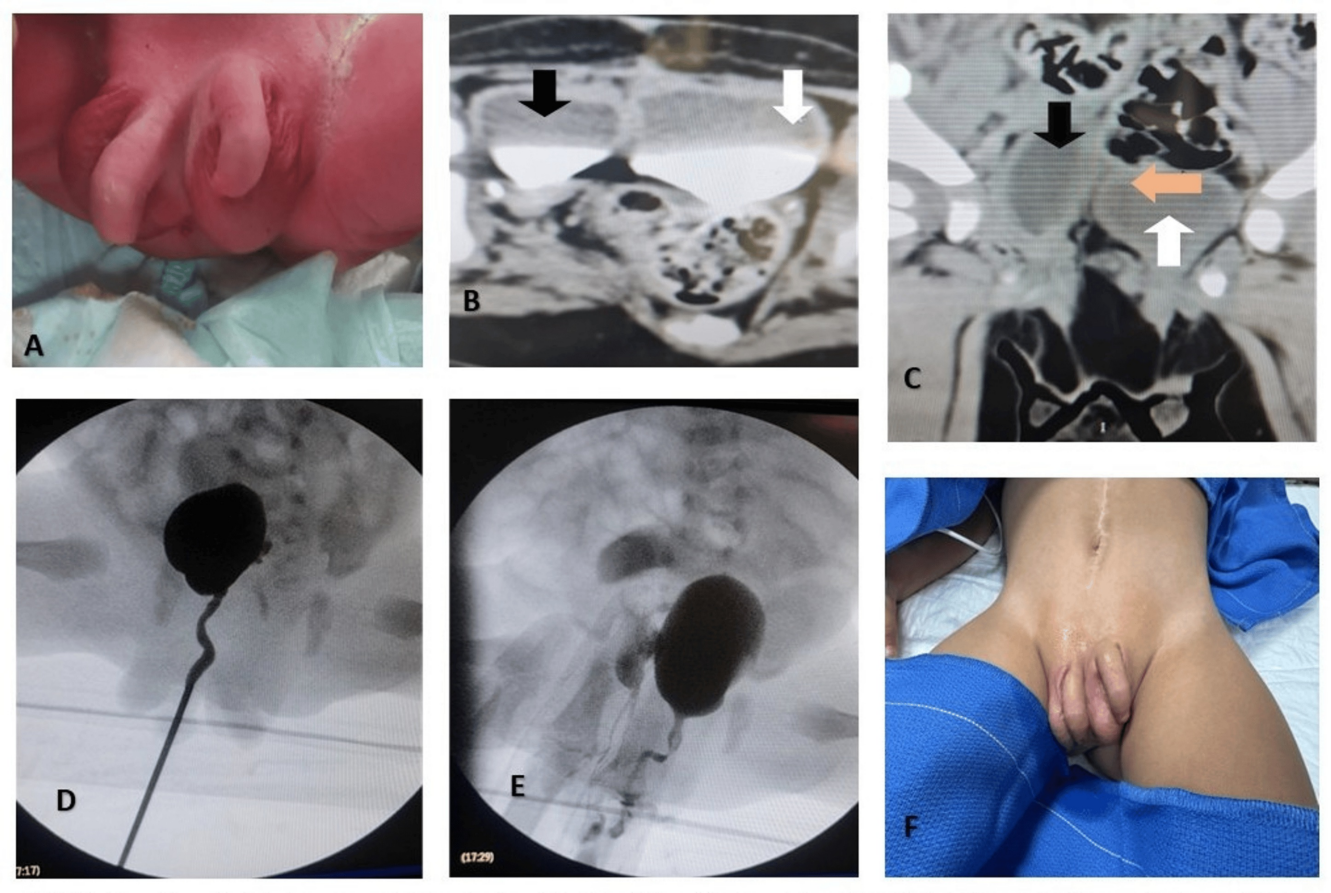
Clinical presentation (A) Genitourinary examination showing diphallia with two fully formed penises, bifid scrotum containing one testis in each hemiscrotal compartment, and duplication of the anal orifice. (B) Axial CT urography in the excretory phase showing duplicated urinary bladders, each receiving drainage from a single ipsilateral simple renal collecting system; right bladder (black arrow) left bladder (white arrow). (C) Coronal CT scan demonstrating bladder duplication with a well-defined septum (orange arrow)  separating the two vesical cavities; right bladder (black arrow) left bladder (white arrow). (D) Right-sided cystogram. (E) Left-sided cystogram. (F) Postoperative recovery after resection of an accessory right colon segment.

A third-gestation male infant was delivered by cesarean section at 37 weeks’ gestation due to maternal hyperbilirubinemia. Prenatal follow-up revealed no exposure to teratogenic agents, adequate folic acid intake, and unremarkable prenatal ultrasonography. There was no family history of congenital malformations or genitourinary anomalies. At birth (Figure 1A), physical examination revealed complete diphallia, a bifid scrotum with a testis in each hemiscrotum, and two anal openings. During the initial assessment, spontaneous micturition was observed from both penises, as well as meconium passage from both anal orifices, predominantly from the left. The remainder of the physical examination was normal. Given the suspected diagnosis and the known associations of this syndrome, consultations were obtained from pediatric neurosurgery and pediatric surgery. Evaluation identified a tethered spinal cord and complete colonic duplication. Renal ultrasonography and uro-CT (Figure 1B) showed normal kidneys, each draining via a single collecting system into a separate urinary bladder. In the pelvis, two fully formed, independent bladders were identified, completely separated in the coronal plane (Figure 1C); the right bladder was smaller in capacity and volume. Over the following days, the infant voided spontaneously and effectively from both urethras and evacuated stool from both anal orifices, predominantly from the left.

A filling cystogram was requested but could not be technically completed in the radiology department; thus, the procedure was scheduled in the operating room. In consensus with neurosurgery, to minimize neural injury related to the tethered cord and reduce the number of anesthetic events, the first surgical step would include spinal cord detethering, preceded by cystoscopy of both systems and cystogram in the same operative session. Cystoscopy and cystogram of the right urinary system revealed a complete and anatomically normal urethra arising from an independent bladder. The external sphincter was barely identifiable, and the prostatic region appeared hypoplastic (Figure 1D). The bladder showed a slightly lateralized ureteral orifice and, notably, floating fecal material. A 2-3 mm fistulous opening was identified on the bladder floor, with fecal discharge through it. Upon maximal bladder filling with contrast medium, passage into the accessory colon was confirmed, with irrigation fluid exiting through the right accessory anus. No vesicoureteral reflux (VUR) was observed during voiding of the right bladder.

The left urinary system demonstrated a complete urethra, normal external sphincter, and better-developed prostate and bladder (Figure 1E). The left ureteral orifice was in Young’s type B position. No VUR was detected. Following cystoscopic assessment, pediatric neurosurgery performed spinal cord detethering during the same operative session. The postoperative course was optimal. After six weeks of recovery, the patient underwent surgical exploration and resection of the right accessory colon, resulting in resolution of the right anal output and the colovesical fistula (Figure 1F). The genitourinary system continued to function well without a need for immediate intervention.

During approximately 18 months of follow-up in pediatric urology, urinary leakage from the right penis was observed during crying or straining, with no leakage from the left penis. Separate videourodynamic studies revealed a hypertonic right bladder with reduced capacity and spontaneous urine leakage even at rest. The left bladder had greater capacity, lower pressures, and minimal leakage (Table 1).

**Table 1:** Videourodynamic findings

Videourodynamic findings	
Videourodynamic study of the right bladder	A urethral pressure profilometry: sphincteric length of 2 cm and a maximum urethral closure pressure of 101 cmH₂O. Filling was initiated at a rate of 10 mL/min. Bladder sensations were absent, and the patient developed incontinence at 15 mL, with Pdet 18 cmH₂O, Pves 44 cmH₂O, and Pura 68 cmH₂O. A second episode of incontinence occurred at 41 mL, with Pdet 6.7 cmH₂O, Pves 93 cmH₂O, and Pura 88 cmH₂O. Diagnosis: Hypertonic bladder (Compliance: 3 mL/cmH₂O). Type III urinary incontinence (Pves 44 cmH₂O). No vesicoureteral reflux observed. Areflexic bladder.
Videourodynamic study of the left bladder	A urethral pressure profilometry: sphincteric length of 3 cm and a maximum urethral closure pressure of 170 cmH₂O. Filling was initiated at a rate of 10 mL/min. Bladder sensations were absent, and the patient developed incontinence at 66 mL, with Pdet 62 cmH₂O, Pves 79 cmH₂O, and Pura 125 cmH₂O. A second episode of incontinence occurred at 73 mL, with Pdet 76 cmH₂O, Pves 121 cmH₂O, and Pura 80 cmH₂O. Diagnosis: Bladder with normal pressures (Compliance: 19 mL/cmH₂O). Type II urinary incontinence (Pves 79 cmH₂O). No vesicoureteral reflux observed. Areflexic bladder.
Renal Function	Serum creatinine: 0.2 mg/dL

Cystoplasty and bladder neck closure

**Figure 2:**
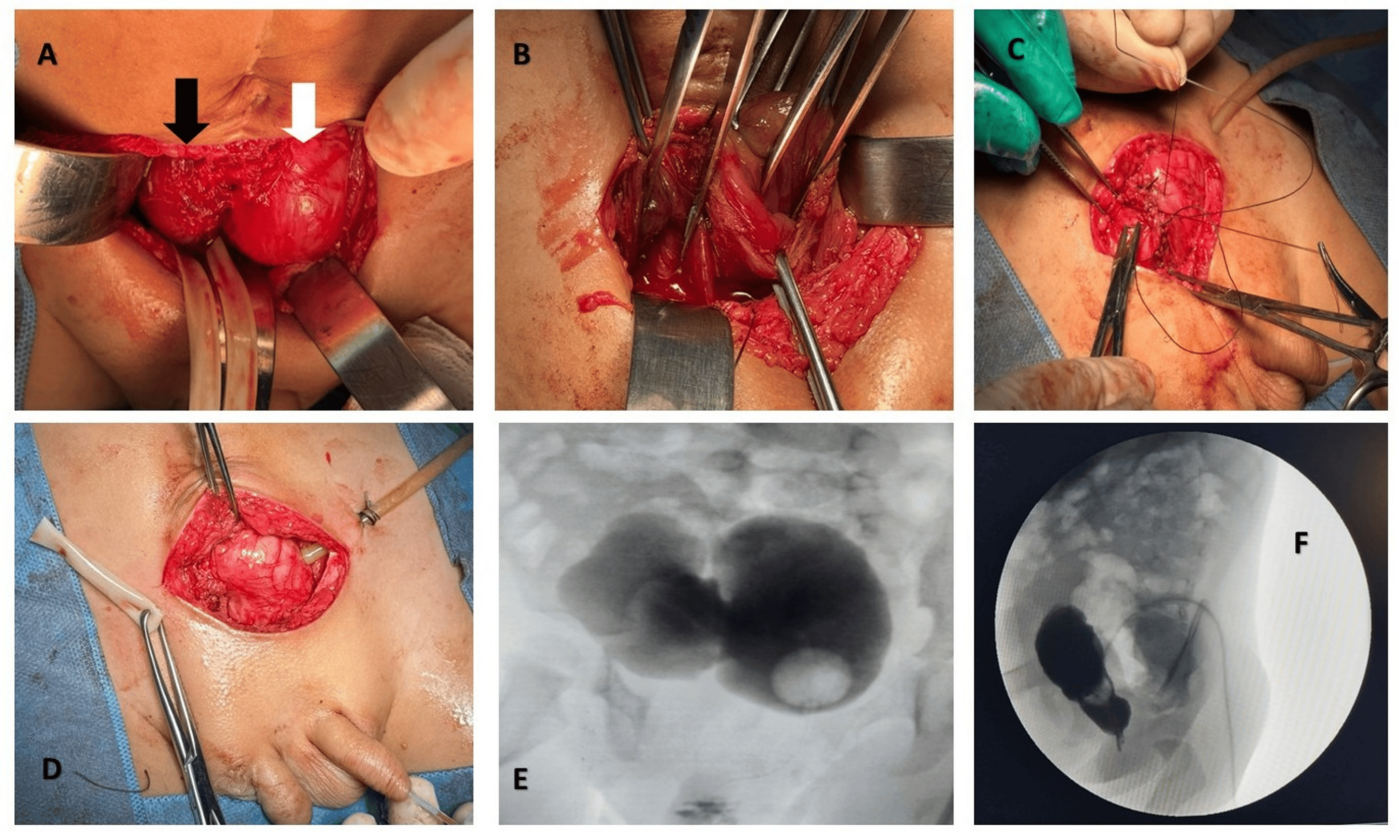
Cystoplasty and bladder slosure (A) Presence of bladder duplication; Right bladder (black arrow) Left bladder (white arrow). (B) Opening of both bladders. (C) Bladder closure. (D) End of surgery. (E) Follow-up VCUG showing adequate bladder filling and emptying, with no evidence of vesicoureteral reflux. (F) Follow-up VCUG, lateral projection.

Based on these findings and multidisciplinary consensus, cystoplasty was performed to unite both bladders without ureteral reimplantation, combined with resection of rudimentary prostatic tissue and closure of the right bladder neck (Figures 2A-2D). Postoperative recovery was satisfactory. A control cystogram at three weeks showed morphologically adequate bladder configuration, absence of VUR, and complete bladder emptying (Figures 2E, 2F). Urinalysis remained normal.

Clinical course

**Figure 3:**
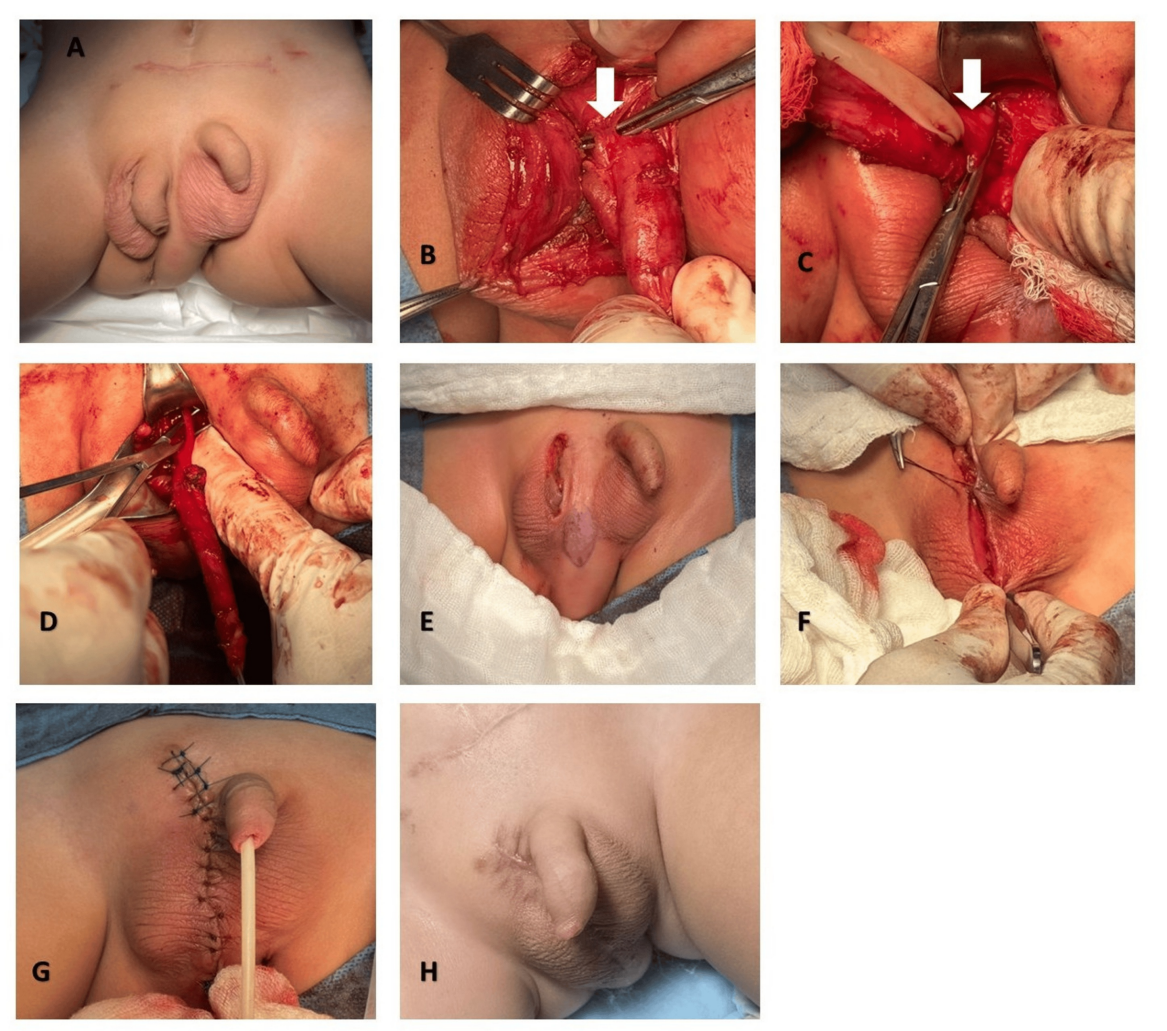
Right phallectomy and scrotoplasty (A) Preoperative image. (B) Release of the suspensory ligament (white arrow). (C) Resection of the corpora cavernosa (white arrow). (D) Urethrectomy. (E) Completion of right phallectomy. (F) Scrotoplasty. (G) Midline closure and completion of the surgery. (H) Current patient status.

Over the following two months, the postoperative evolution was satisfactory. The patient was then scheduled for right accessory phallectomy with posterior urethrectomy and scrotoplasty (Figure 3). Recovery was favorable, with the only postoperative complication being a minor scrotal seroma, which was successfully managed with conservative measures. The patient is currently able to walk, and defecation occurs exclusively through the left anal orifice. From a urinary standpoint, given the patient’s age, voiding control is still under development. However, he demonstrates spontaneous urinary output without incontinence and is awaiting a follow-up urodynamic study. Written informed consent was obtained from the patient’s parents for all surgical procedures, clinical photography, and open-access publication of this case. Institutional review board approval was not required for this single case report, in accordance with local institutional policy.

**Figure 4:**
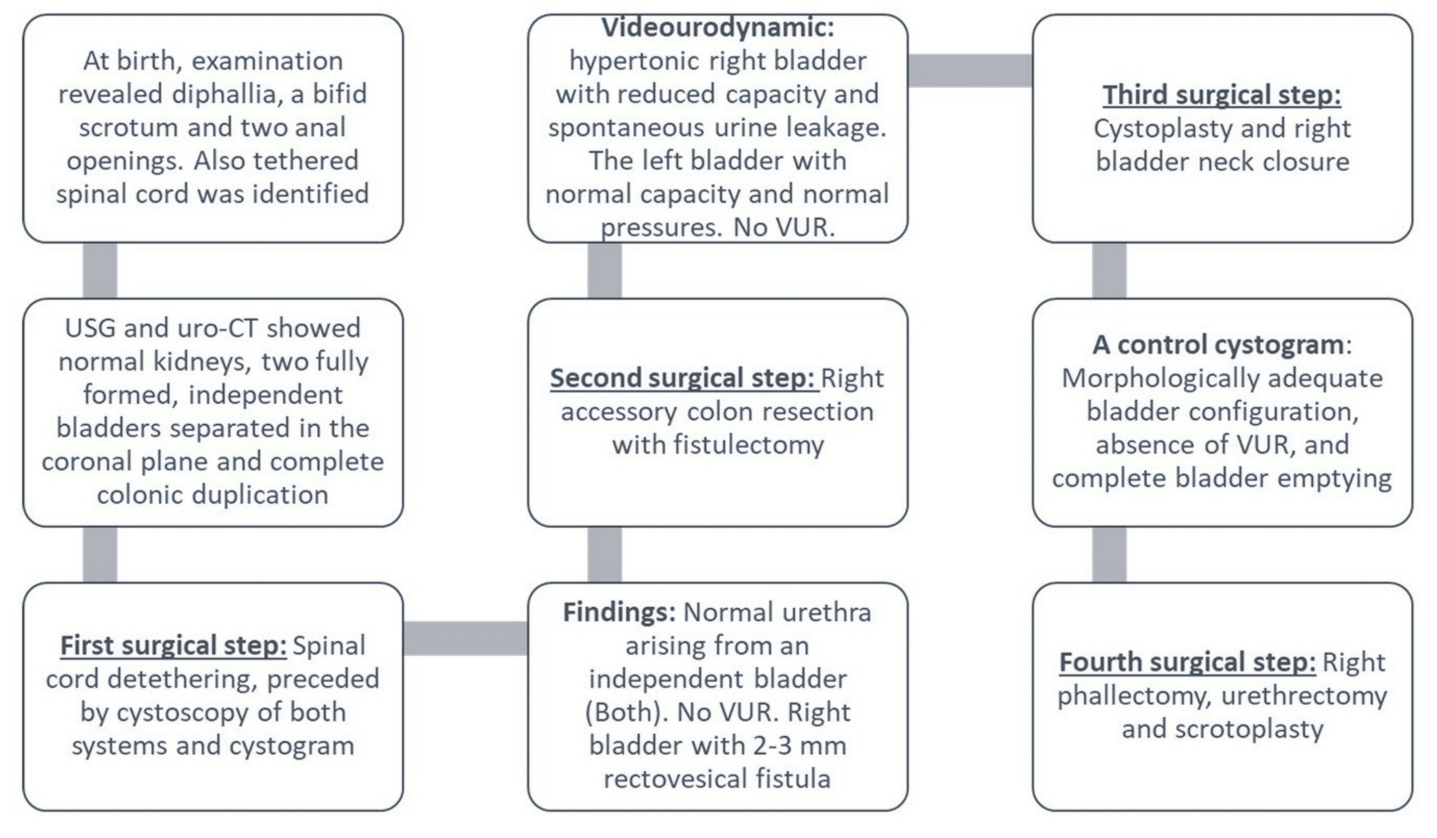
Timeline summary of the patient’s clinical course

Figure 4 demonstrates the timeline summary of the clinical course of the patient.

## Discussion

This case illustrates a highly uncommon variant of caudal duplication syndrome, characterized by complete duplication of the bladder, urethra, prostate, penis, scrotum, colon, and spinal cord involvement, with a colovesical fistula that was not identified until cystoscopy and cystogram were performed. While previous authors such as Kuang et al. [1] and Wang et al. [3] have emphasized the variability of phenotypic expression in CDS, the present case reinforces that even anatomically unanticipated anomalies may be present despite initial imaging appearing unremarkable. This diagnostic limitation supports the need for systematic endoscopic and urodynamic assessment before defining the sequence of surgical interventions [10].

The management strategy in this case aligns with the growing consensus in the literature that CDS treatment must be individualized and staged [3,6]. Wang et al. [3] and Radu-Iulian et al. [7] highlight that no standardized surgical algorithm exists, and that the therapeutic approach should be driven by functional priorities rather than anatomical replication alone. In our patient, early spinal cord detethering and colonic fistula resolution were prioritized over urological reconstruction, reflecting the principle that neural and gastrointestinal risks often precede reconstructive urology in urgency. This sequencing approach is consistent with the multidisciplinary strategy described in the experiences of Delcont et al. [2] and Liu et al. [9].

The decision to avoid ureteral reimplantation or cystectomy and instead perform selective cystoplasty was based on functional urodynamic data rather than purely anatomical considerations. This is aligned with the contemporary shift toward functional preservation and minimally invasive intervention whenever possible, as reported by Wisenbaugh et al. [8]. This management choice aligns with the contemporary understanding that, in caudal duplication syndrome, surgical interventions should aim to preserve function and optimize long-term outcomes rather than pursue anatomical normalization alone. Nevertheless, it is important to recognize that in certain patients, bladder duplication may coexist with normal renal function as well as preserved voiding and continence mechanisms; in such cases, conservative management and close follow-up may represent the most appropriate approach [10-12].

Once functional stability had been achieved, the final stage of management focused on aesthetic and anatomical reconstruction. A right-sided phallectomy with urethrectomy was performed, based on functional asymmetry between the duplicated systems. The right urinary tract demonstrated poor bladder compliance and had previously undergone bladder neck closure, rendering it nonfunctional for voiding. Phallectomy was intentionally delayed until neurological, gastrointestinal, and lower urinary tract stability had been achieved, allowing reconstruction to be performed in a controlled setting without compromising continence or neurological outcomes. This procedure was complemented by scrotoplasty to restore external genital morphology, preserve symmetry, and minimize potential long-term cosmetic and psychosocial impact. Beyond its technical complexity, phallectomy in pediatric patients raises important ethical considerations, particularly when the indication is primarily reconstructive rather than life-saving. Previous reports on diphallia emphasize that management involves not only surgical planning but also careful ethical deliberation, given the rarity of the condition, uncertainty regarding long-term outcomes, and the irreversibility of genital procedures performed before the patient can participate in decision-making [6,7]. Ethical frameworks addressing congenital genital anomalies underscore the need to balance beneficence and non-maleficence with respect for the child’s future autonomy, applying a best-interest standard supported by comprehensive parental counseling and informed permission [13]. In the present case, decision-making was multidisciplinary and also incorporated the parents’ informed preference to proceed with phallectomy after extensive discussion of functional, cosmetic, and potential psychosocial implications.

Long-term follow-up remains essential, as emphasized consistently across previous literature. Given this patient’s preserved spontaneous voiding from the bladder and absence of vesicoureteral reflux, the urinary prognosis appears favorable; however, continence, bladder compliance, and later reproductive potential will need to be reassessed throughout growth. Follow-up must be individualized and maintained by each specialty throughout the patient’s development [3].

## Conclusions

This case highlights an exceptionally rare and functionally complex presentation of caudal duplication syndrome, emphasizing that even unanticipated anatomical variants may be present despite normal initial imaging. Optimal management requires an individualized, staged, multidisciplinary, and function-oriented approach. Early involvement of all relevant specialties and strategic coordination of surgical interventions are essential to reduce procedural burden. Management should be prioritized according to functional urgency, supported by urodynamic assessment of the genitourinary tract and favoring minimally invasive, organ-preserving strategies whenever possible. Long-term, individualized follow-up by all involved specialties is critical to optimize outcomes throughout growth.
